# Assessing emergency medical care in low income countries: A pilot study from Pakistan

**DOI:** 10.1186/1471-227X-8-8

**Published:** 2008-07-03

**Authors:** Junaid A Razzak, Adnan A Hyder, Tasleem Akhtar, Mubashir Khan, Uzma R Khan

**Affiliations:** 1 Department of Medicine, Aga Khan University, Karachi, Pakistan; 2 Department of International Health and Center for Injury Research & Policy, Johns Hopkins Bloomberg School of Public Health Baltimore, USA; 3 Fatima Memorial System, Lahore, Pakistan; 4 Pakistan Medical Research Council Islamabad, Pakistan

## Abstract

**Background:**

Emergency Medical Care is an important component of health care system. Unfortunately it is however, ignored in many low income countries. We assessed the availability and quality of facility-based emergency medical care in the government health care system at district level in a low income country – Pakistan.

**Methods:**

We did a quantitative pilot study of a convenience sample of 22 rural and 20 urban health facilities in 2 districts – Faisalabad and Peshawar – in Pakistan. The study consisted of three separate cross-sectional assessments of selected community leaders, health care providers, and health care facilities. Three data collection instruments were created with input from existing models for facility assessment such as those used by the Joint Commission of Accreditation of Hospitals and the National Center for Health Statistics in USA and the Medical Research Council in Pakistan.

**Results:**

The majority of respondents 43/44(98%), in community survey were not satisfied with the emergency care provided. Most participants 36/44(82%) mentioned that they will not call an ambulance in health related emergency because it does not function properly in the government system. The expenses on emergency care for the last experience were reported to be less than 5,000 Pakistani Rupees (equivalent to US$ 83) for 19/29(66%) respondents. Most health care providers 43/44(98%) were of the opinion that their facilities were inadequately equipped to treat emergencies. The majority of facilities 31/42(74%) had no budget allocated for emergency care. A review of medications and equipment available showed that many critical supplies needed in an emergency were not found in these facilities.

**Conclusion:**

Assessment of emergency care should be part of health systems analysis in Pakistan. Multiple deficiencies in emergency care at the district level in Pakistan were noted in our study. Priority should be given to make emergency care responsive to needs in Pakistan. Specific efforts should be directed to equip emergency care at district facilities and to organize an ambulance network.

## Background

Health care in developing countries has not traditionally focused on emergency medical care [1]. Paradoxically, greater attention is needed in developing countries, where injury rates are higher and fewer injury control activities have been undertaken [2]. Emergency care can make an important contribution to reducing avoidable deaths and disability, especially in low-and middle-income countries [3]. But the state of health care in Pakistan indicates that the public health system has never focused on emergency medical care. Data from seven developing countries show that 14 of 21 hospitals lacked an adequate system for triage and method for initial patient assessment leading to delayed treatment [4].

Pakistan is a developing country in need of effective emergency medical care [5,6]and data from Pakistan clearly indicates this need. According to a study on the burden of disease in Pakistan diarrhea, lower respiratory infections, ischemic heart disease, septicemia, and injuries are among the top 15 causes of premature deaths [7]. Data from Karachi, the biggest city of Pakistan show that the main causes of death in adults included circulatory disorders, injuries (road traffic crashes, burns) and complications of pregnancy [8]. A study conducted in rural northern Pakistan found that that the reason for poor outcome in many cases of surgically treatable illness included misinterpretation of severity of symptoms by first level providers and mis-triage from the first level facility [9]. Another study showed that the majority of children who died were actually taken to a health care facility immediately at the onset of disease; however longer time to get to referral facility was cited as the most important cause of these deaths [10]. A study also reported less than half of the households who experienced serious illnesses were taken to a nearest first-level care facility mainly because of dissatisfaction as reported by participants [11].

Emergency medical care has not been part of health sector reform efforts at the national or state level in Pakistan. At the same time, emergency medical care has also been neglected in health research in the country. As a result, the only form of emergency care that has been available throughout the nation, especially in larger hospitals and clinics, is based on post-colonial models of emergency departments in the public sector. Though in recent years, non-profit organizations have also established emergency transport services in selected urban centers in the country, such services are limited and restricted to very few areas [12].

Emergency care needs to be well planned and supported at all levels of care, from the occurrence of an acute medical event in the community to the provision of appropriate care at the hospital [3]. Therefore, as a first step towards planning a national model for emergency care, we carried out a pilot study and situation analysis of the existing system of emergency care. It included the assessment of the structures, people and processes in place in two districts of the country that would provide a snap shot regarding the state of emergency care and enable the piloting of country-specific instruments for performing such assessment. This paper presents the results of the pilot study in the initial two districts.

### Health Care provision in Pakistan

Health care provision in Pakistan comprises both public and private services. The private sector serves nearly 70 percent of the population, and is primarily a fee-for-service system, though the share of such private sector in emergency care of Pakistan is not known. The main source of funding of the public sector is the Government and comprises more than 10 000 facilities ranging from Basic Health Units (BHUs) to tertiary referral centers (Table 1). Dispensaries and BHUs provide outpatient services and each BHU covers around 10 000 population. Maternal Child Health Centers (MCHCs) provide basic maternal and child care, including ante-natal care, nutrition, normal delivery and immunization. Rural Health Centres (RHCs) provide more extensive outpatient services and some inpatient services, and each RHC covers around 30 000–45 000 population. In Pakistan, both BHUs and RHCs are called "primary health care" units (Table 1). The Tehsil (sub-district) Headquarters Hospital covers 100,000–300,000 population at sub district level with 40 to 50 beds; while the District Headquarters Hospital serves a geographical district of about one to two million people with 80–100 beds [13].

**Table 1:** General Description of Services at Public Health Care Facilities in Pakistan.

**First Level Facilities/Primary Care Facilities**	**Referral Level Care Facilities**	**Tertiary Care Facilities**
**Dispensaries, Mother and Child Health Centers, Basic Health Units:**	**Tehsil Headquarter Hospital (THQ):**	**Tertiary Care Facilities**
Primarily outpatient curative and preventive services	Tehsil Headquarter Hospital (THQ):	-Teaching Hospitals
-Has limited staff (one doctor or lady health visitor and/or a dispenser)	-Outpatient and Inpatient services	-Sub-specialty care
-Opens during day time, serves 10,000–20,000 population	-40–150 beds, x-ray, laboratory, surgical facilities available	-Mainly located in large urban centers
	-At least 3 specialists: Obstetrics/Gynecologist; Pediatricians, General Surgeon	
	-Serves 0.5–1 million population	
		
**Rural Health Center:**	**District Headquarter Hospital/Civil Hospital:**	
-Outpatient and some Inpatient Services	-Extensive outpatient and inpatient services	
-Provides basic emergency care	-100–150 beds; Eight or more specialists	
-10–20 beds, x-ray, laboratory and minor surgical facility	-Serves 1–2 million population	
-Serves 50,000–100,000 population		

## Methods

### Study Design

The study consisted of three cross-sectional assessments conducted between August to September 2004. The scientific and ethical committee of the Pakistan Medical Research Council approved the study.

### Study Setting

The facility survey was carried out in two districts of Pakistan, a low income country with a per capita income of less than US$ 720 (World Bank) and a health spending of about 0.8% of GDP [14]. Data from primarily rural facilities were collected from one of the districts (Peshawar), while urban facilities were studied from the other district (Faisalabad). The district Peshawar has a population of 2,038,629 (1998 census) with 988,005 classified as urban and 1,050,624 as rural. Males constitute 52% of the population (males 1,060,087 and females 978,542) and the district has 74 rural health facilities. A convenience sample of rural health facilities was selected in 5 categories: large civil hospitals, Rural Health Centers (RHCs), Basic Health Units (BHUs), Dispensaries (pharmacies), and Maternal Child Health Centers (MCHCs). The criterion for inclusion was a fully functioning health facility conveniently accessible to study staff. Out of 74 functioning rural health facilities in Peshawar, 22 (30%) were selected as shown in Table 2.

**Table 2:** Health facilities studied in districts of Peshawar and Faisalabad in Pakistan; August-September 2004.

**T**ype of Health Facility	**P**eshawar		**F**aisalabad	
		
	Total Number	Number included in study	Total Number	Number included in study
District headquarter Hospital	01	01	01	01
Taluka headquarter Hospital	0	0	04	04
Rural Health Centre	04	03	11	00
Basic Health Unit	56	14	168	03
Maternal & Child Health Centre	01	01	06	02
Dispensaries	12	3	108	10
**Total**	**74**	**22**	**298**	**20**

The district Faisalabad has a population of 5,430,000 (census 1998) with 2,319,000 being urban and 3,111,000 being rural. More than half (52%) of the population are males (male 2,823,600 and female 2,606,400). A total of 20/298 (7%) facilities from urban locations in Faisalabad including District Headquarters, Sub-district [Taluka] Headquarters hospitals, Basic Health Units, Maternal and Child Health Centers and Government city Dispensaries were selected on convenience sampling basis as shown in Table 2.

### Selection of Participants

The three cross-sectional assessments focused on selected community leaders, health care providers, and health care facilities were conducted. All the participants were selected based on convenience sampling from the same areas where health care facility assessments were done. Community leaders included religious scholars, school principals, local government officials [called *nazims*] and city council members.

For the second assessment, health care providers were selected from the facilities chosen for the third part of the study on convenience basis. Health care providers include medical superintendents, health officers, lady health visitors (registered paramedical personnel), and dispensers (pharmacist) were interviewed. Facility incharge officers were interviewed for facility assessment.

### Methods of Measurement

As part of the study, three data collection instruments were designed by the investigators. A literature search did not identify a standardized and universally accepted instrument specifically for emergency care assessment in a low income country. Thus instruments were created with input from existing models for facility assessment such as those used by organizations like the Joint Commission of Accreditation of Hospitals [15] and the National Center for Health Statistics in USA [16], and the Pakistan Medical Research Council [17]. Data collection instruments were then translated in Urdu (Pakistan's national language) and pre-tested in a separate area prior to use. Three open ended forms were created for this study. The first was a 2-page questionnaire including 11 items. It addressed community leader's perception of health care facilities and emergency care, their expectations, problems and recent experiences with the emergency system. The second form consisted 29 items (4-pages) evaluating types of emergencies commonly seen by practitioners, their training for the management of these emergencies, difficulty in transfer of patients to higher facilities, and barriers to provision of emergency medical care.

For assessing health care facilities information was obtained from the facility incharge (director), by interviewer assessment during the facility tour, and by a review of patient logs. This included 8-pages with a 59 items questionnaire and an item-by-item inventory to identify drugs, fluids, and clinical supplies in the facilities. Patient logs were used for the number of patients visiting emergency and out patient department. Face to face interviews were conducted from community leaders, health care providers and in charge of the facility.

### Data collection and processing

Data was collected by senior staff of the Pakistan Medical and Research Council (PMRC), a research body of Pakistan. PMRC personnel were trained during this project. The data collectors were not blinded regarding the purpose of the study. As per approval from PMRC, and their usual survey practice, verbal informed consent was sought from each participant. The filled questionnaires were sent to the PMRC head office in Islamabad where data entry and consistency checks were performed in Microsoft Excel (Microsoft Corporation, Redmond, WA).

### Primary Data Analysis

Data analysis was performed using Statistical Package for the Social Sciences (SPSS, Inc., Chicago, IL). It involved separate estimation of simple frequencies for each component of the study. Due to the pilot and small nature of the study, advanced analysis was not considered appropriate.

## Results

### Community Survey

Out of 44 community leaders, 20 (46%) were from urban areas and 24 (54%) from rural areas of the districts. The majority of respondents were male 36/44 (82%), more than half were less than or equal to 50 years of age 13/44 (57%) and nearly all married 43/44 (98%). 91% (40/44) participants were not satisfied with the overall performance of healthcare facilities and even more – 98% (43/44) – were not satisfied with emergency care provided. The most common reasons for dissatisfaction with overall health care facilities were (n = 40): lack of perceived proper care 15 (38%), lack of facilities 16 (40%), and non-availability of medical officers 9 (23%). The most common reasons for their dissatisfaction with the emergency care provided were (n = 43): lack of perceived proper emergency care 22 (51%), and lack of medicines 18 (42%).

The majority of participants 36/44 (82%) mentioned that in the event of a health related emergency they will not call an ambulance. The most common reason stated was that the ambulance service does not function properly in the government system 22/36 (61%). In response to a question on their preference for a healthcare facility in case of a future emergency, 24/44 (55%) mentioned that they would take the patient to a hospital, 10/44 (23%) preferred to visit the District Headquarter hospital, and 9/44 (21%) preferred the Sub-district (Taluka) Headquarter hospital. In case of emergency involving children, 15/44 (34%) of respondents preferred a private hospital and 11/44 (25%) preferred the District Hospital. Similarly, in case of a health emergency involving women, 18/44 (41%) preferred a private hospital and 11/44(25%) preferred the District Hospital.

The most common set of expectations by the participants during a healthcare visit in an emergency were reported to be: competent emergency care staff 27/37(73%), and free availability of medicines 10/37 (27%). At the same time, responses on the most common problems in the emergency care system in the area were: lack of equipment 24/43 (56%), lack of ambulance system 21/43 (49%) and substandard services 10/43 (23%). While responding to the request for suggestions on improving the emergency care system; 29/44 (66%) mentioned the importance of communication and an organized network of ambulances; and 26/44 (59%) emphasized the importance of fully equipped emergency units.

These community leaders were asked about their most recent experience with the emergency system in their district. 29 respondents provided the following feedback: a hospital ambulance had been used by only 2/29 respondents (7%) and the rest had either used their own transport or hired a vehicle for transferring a patient to a health care facility. About 26/29 (90%) of all the transported patients were reported to have reached the health care facility within an hour. The majority of these cases were attended by physicians in the emergency department 23/29 (79%), and the rest were attended by paramedics. The expenses on emergency care for the last experience were reported to be 5,000 Pakistani Rupees or less (equivalent to US$ 83) for 19/29 (66%) respondents.

### Health Care Provider Survey

A total of 44 health care providers were surveyed; 20 (45.5%) from urban, 24 (54.5%) from rural areas. Out of total respondents; 30/44 (68%) were physicians, 6/44 (14%) dispensers, 4/44 (9%) paramedics and 3/44 (7%) Lady Health Visitors (respondent designation was missing in 1 case). More than half 38/44 (52%) were 50 years or less of age and the majority were males 39/44 (89%). When asked if they had received any post graduate medical education, 37/44 (84%) said they have not. More than two thirds 35/44 (80%) were working in the health facility for 6 years or less, and13/44 (30%) were there for 2 years or less.

More than half providers 26/44 (59%) thought that management of medical emergencies was part of their duties. The most common emergencies reported by health care providers were (in rank order): Dehydration/Diarrhea/Vomiting 19/44 (43%) followed by Fever 16/44 (36%), Injuries 15/44 (34%) and Respiratory diseases 12/44 (27%). When asked if they thought that their facilities were adequately equipped to treat emergencies, 43/44 (98%) responded in the negative. The most often cited reasons for poor emergency care capacity were lack of proper equipment 40/44 (91%), followed by lack of life saving drugs 26/44 (59%), lack of trained staff 26/44 (59%), lack of oxygen 16/44 (36%), and lack of operating rooms 11/44 (25%). When asked if they had received adequate training in emergency care during their education, the majority answered positively 30/44 (68%). These providers ranked the following emergencies as most difficult to handle by their facility: 1- Injuries (especially Head Injuries 23/44 or 52%, Road Traffic Injuries 22/44 or 50%, Burn injuries 18/44 or 41%, and Firearm injuries 7/44 or 16%), 2- Stroke 22/44 (50%), 3- Myocardial Infarction 22/44 (50%), and 4-Dehydration 20/44 (45%). 19/44 (43%) providers thought that it was very difficult (or difficult) to transfer a patient to a higher level facility.

### Facility Assessment

In the survey of healthcare facilities (n = 42); 20 (48%) were urban health centers and 22 (52%) were rural centers. These sites included a mix of facilities including health centers, dispensaries, and hospitals as shown in Table 2. All these facilities were within 10 kilometers distance from major roads. Almost three quarter 31/42 (74%) of facilities had no budget allocated for emergency care (information for five facilities was missing). Most of these facilities 41/42 (98%) charged user-fee. The majority of funds for emergency care were being consumed for antibiotics 19/42 (45%) and analgesics 19/42 (45%). Only 2/42 (5%) facilities had backup surgical support, while 3/42 (7%) facilities had backup anesthesia coverage and 6/42 (14%) facilities had operation theater staff, while none had a surgical intensive care unit or an orthopedic surgeon.

The patients seen in the Out Patient Departments (non-emergency units) of these facilities varied from 5 per day to more than 936 per day. Only 8/42 facilities (19%) were accessible after routine working hours. Twenty facilities (60%) had formal emergency departments while 17 (40%) lacked any designated area for emergency care. Less than half (n = 20) reported that the number of visits from the monthly census ranged from 200 to 999 patients. During the last one month, in the emergency department the number of visits per day ranged from 0 to 51 for men, 5 to 100 for women, and 0 to 60 for children less than or equal to 15 years old. Table 3 shows the most common reasons to visit emergency department during the last one month in these facilities which were: skin infections, respiratory infections, fever, diarrhea or dysentery, gastroenteritis, backache and cough/flu.

**Table 3:** Common reasons for visit to the health facility (n = 42; patient log) in Pakistan, August-September 2004.

**E**mergencies	**P**ercentages (%)
Respiratory Tract Infection	66.7
Fever	64.3
Diarrhea and Dysentery	54.7
Gastroenteritis	50.0
Backache	31.0
Flu/Cough	28.6

(Note: since individuals come with multiple complaints to the health facility, the rows total does not add to 100%)

A review of medications and equipment available showed that many of the critical supplies needed in an emergency were not found in these facilities (Table 4). On the other hand some facilities did have antibiotics and intravenous fluids which are important for the management of septic patients. Table 4 shows pain medications were the most widely available medication category, while oxygen and airway supplies were available in higher order facilities.

**Table 4:** Physical resources at health facilities in Peshawar and Faisalabad, Pakistan; August–September 2004 (n = 42).

***Supplies***	BHU/MCHC* *n = 33(%)*	RHC* *n = 3 (%)*	DHQ/THQ* *n = 7 (%)*
**Oral Medicines**			
Aspirin	8 (24)	2 (67)	5 (71)
Oral Antibiotics	28 (85)	2 (67)	6 (86)
Nitroglycerine	0	0	3 (43)
Pain Medications	29 (88)	3 (100)	6 (86)
Albuterol nebulized	5 (15)	2 (67)	4 (57)
Anticonvulsants	8 (24)	0	5 (71)
			
**Injectables**			
IV fluids	24 (73)	3 (100)	6 (86)
IV Antibiotics	23 (70)	2(67)	5 (71)
IV Catheter/Cannulas	1 (3)	1 (33)	3 (43)
Oxytocin	0	0	3 (43)
Tetanus Toxoid	12 (36)	3 (100)	6 (86)
Antivenin	0	0	1 (14)
			
**Airway/Breathing**			
Oxygen	0	1(33)	5(71)
Ambu Bag with Mask	0	2(67)	4(57)
Oral Airway	1(3)	0	2(29)
Nasal Airway	0	0	2(29)
Endotracheal tube	0	0	2(29)
			
**Cardiac Emergency**			
EKG Machine	1(3)	1(33)	4(57)
Defibrillator	3(9)	1(33)	1(14)

*BHU/MCHC = Basic Health Unit/Mother and Child Health Center

RHC = Rural Health Center

DHQ/THQ = District Headquarter Hospital/Tehsil Headquarter Hospital

## Discussion

This study is one of the first attempts to perform a limited situation analyses of the emergency medical care system in two selected districts of Pakistan. The intent is to provide a pilot evaluation of the existing situation that can guide efforts to improve the quality of care, and outcomes, for patients arriving in hospital and to apply new tools developed for this purpose. This study shows poor public perception of emergency care by the users and this is similar to findings by another study on health services in general in Pakistan [11]. The main reasons for this low satisfaction mentioned in this study included poorly equipped facilities, lack of capable staff, and lack of proper care.

Questions relating to suggestions for improvement of emergency care were consistently answered and community leaders emphasized the importance of fully equipped emergency rooms and the need for an organized network of ambulance services. Ambulances were used very infrequently while private vehicles were available for emergency transport in a large number of cases. This also translated to a perception of poor reliability of ambulance service as a majority of users thought that ambulance service does not function properly in the government system. These results are consistent with previous work in Karachi, as most people do not use ambulances in case of a medical emergency [18]. Despite the lack of proper ambulance service, however, the majority of users reported that they were able to reach a health care facility within one hour. This represents a good process measure but might not representative of other districts in Pakistan, especially those in highly urban or remote areas.

The findings from community leaders were also consistent with the impressions of health care providers who also did not think that their facilities were adequately equipped to treat emergencies. They identified equipment, medications (including oxygen), untrained paramedic staff, and lack of operation facilities as major weaknesses in the emergency care system. A large number of physicians feel that they were facing major difficulties in referring a patient to a higher level of facility due to poor transportation and lack of proper communication systems. While more than half of the providers suggested that they had been trained for emergency care, it is clear that the community does not perceive providers to be well trained to deliver such care. The basic five year medical education program in the public sector of Pakistan is accredited by the Pakistan Medical and Dental Council [19]. The curriculum does not provide specific competencies in emergency medicine and rotations in the emergency room are not required of all medical students. Training could be focused on common emergencies seen in such places, and may represent a useful point of intervention for the medical profession to improve emergency care. A number of studies have reported a beneficial impact from specialized emergency training. For example in Trinidad, improvement in trauma patient outcome has been reported post Advanced Trauma Life Support training with a decrease in mortality (67% vs. 34%) among most severely injured patients [20]. In Malawi, improved triage and emergency care for children also reduced inpatient mortality from 10–18% before the changes were made (before 2001) to 6–8% afterwards [21].

The facility assessment showed that generally these health outlets were neither designed nor equipped to handle emergencies. Basic emergency resuscitation items such as oxygen and essential drugs were not present, and EKG machines and defibrillators were almost non-existent. Equipment for airway management, the first step in the emergency care of a patient was also not available. Studies done in other developing countries such as India, Ghana, Vietnam, and Mexico also show shortages of airway equipment, chest tubes, and trauma-related medications [22]. This identifies a supply side intervention – provision of equipments that could be better supplied to improve outcome. This study did not focus on the management of specific types of emergencies or the costs of emergency care in Pakistan. However, it is evident that the management of emergency care is part of the overall administration of each facility and dedicated units are rare.

The study only obtained information from 42 facilities and this small sample makes it difficult to further categorize data based on the level or the location of the facility. The convenience sample also introduces other issues such as the fact that selected facilities were close to each other, therefore may be different from more remote locations and thus limit the generalizibility of the study. This study evaluated only the government-run systems but not private facilities, where a considerable portion of the population receives usual health care. However, in Pakistan, the government system takes care of most emergency cases, especially for those who are lower income. Bias due to self report might also affect our results since we used an interview approach and many of the presenting complaint questions are affected by the interpretation of the person being interviewed. There are expected differences in level of selected study health facilities for example the scope of dispensaries and BHUs are only outpatient non-emergency curative services but were included in the assessment of physical resources. Their physical resources therefore may meet the planned activities of caring for stable out- patients.

Community leaders were selected based on a convenience sample. It is possible that due to their respective position in their community they enjoy quicker and better health care access and therefore their experiences and perception may not represent rest of the population. On the contrary, it is also possible that their status in the community exposes them to issues and problems of the wider community and therefore their responses could be more representative of the experience of the larger population. A population based survey of a random sample would be needed to confirm or refute these assumptions in the future. Despite these limitations, this study provides a first step in improving emergency medical care.

The study has contributed both methods and an opportunity for capacity development of research personnel in Pakistan. The instruments developed for this pilot are now being modified and reviewed in line with other international documents for further application in a larger study [23] The collection of standardized data, specific analysis from 3 different target groups, and cooperation across public and private research institutions in Pakistan were important capacity development features of this study. Work on this project has also enables critical input into other training initiatives in Pakistan such as those supported by the National Institutes of Health, USA [24].

## Conclusion

Developing countries like Pakistan have a high burden of disease that needs emergency medical systems [25]. At the same time, the state of emergency care in such countries is sub-optimal, especially in the public sector. There need to be some concerted effort to improve the state of the emergency system especially in low and middle income countries. This study calls for a more systematic and representative study of emergency care in Pakistan, and a national dialogue on the role of emergency services within the Pakistani health system. It is time for health systems to confront the challenge of EMC.

## Competing interests

The authors declare that they have no competing interests.

## Authors' contributions

JAR was involved in conception of the study, study design, data analysis and paper writing. AAH was involved in conception of the study, technical assistance with study design and paper writing. URK was involved in data analysis and paper writing. TA and MK were involved in study design, data collection and quality control

## Pre-publication history

The pre-publication history for this paper can be accessed here:


